# The paediatric global musculoskeletal task force - ‘towards better MSK health for all’

**DOI:** 10.1186/s12969-020-00451-8

**Published:** 2020-07-14

**Authors:** Helen E. Foster, Christiaan Scott, Carl J. Tiderius, Matthew B. Dobbs

**Affiliations:** 1 Newcastle University, UK and Newcastle University Medicine Malaysia, Newcastle upon Tyne, UK; 2 University of Cape Town, Cape Town, South Africa; 3 Skane University Hospital, Lund, Sweden; 4 Washington University School of Medicine, St Louis, USA

**Keywords:** Musculoskeletal, Global, Access to care, Awareness, Workforce

## Abstract

There is increasing concern about the emerging global non-communicable diseases (NCDs) burden. The focus has mainly been on NCDs in adults but it is important that MSK morbidity in both children and adults is included in strategic planning. There have been considerable advances in the understanding and treatment options for children and young people (CYP) and clinical outcomes are improving for those who can access such high quality care. However vast inequity exists and there are many CYP who live in areas of the world with high burden of health care challenges, compounded by paucity of specialist care and limited access to treatments. The Paediatric Global Musculoskeletal Task Force aims to raise awareness about unmet needs for CYP with MSK conditions, promotion of MSK health through lifestyle and the avoidance of injury. We aim to leverage change through ‘working together better’.

## Background

There is increasing concern about the emerging global burden of non-communicable diseases (NCDs) and the contributory impact of musculoskeletal (MSK) morbidity on NCDs such as dementia and cardiovascular disease [1–3]. MSK health is often under prioritised in health care systems geared towards the burden of communicable diseases or NCDs in adults [2]. There is also the burgeoning tide of obesity as a harbinger of ill health, including MSK morbidity across the life course, and the impact of chronic pain which often has origins in childhood. The ongoing COVID-19 global pandemic has exposed fault lines in health systems all over the world and also brought into sharp focus global inequalities in the delivery of health care.

The needs of children and young people (CYP) and especially for those with MSK problems, are often overlooked - more so in countries with limited resources and paucity of specialists [4, 5]. Globally, there are many CYP (likely millions) with MSK conditions; the majority living in the poorest and most populous countries in Asia, Africa and South America [5, 6]. Delay in access to ‘right care’ is well reported [7] and increasingly important given advances in treatment options; the disparity is stark with worse clinical outcomes reported in countries with limited resources [8, 9]. The paucity of workforce capacity in the most populated and least resourced countries [10] further adds to the challenges and many CYP are unlikely to receive equivalent care to those living in well-resourced countries [11, 12].

The Paediatric Task Force for Global Musculoskeletal Health (‘Task Force’) is a virtual global community with a shared vision to ‘work better together’ to improve the lives of CYP. Our focus is ‘MSK’ in the broadest sense and optimising MSK health through lifestyle, avoidance of injury (including road safety) and reducing obesity. The Task Force was set up in 2017 as part of the Global Alliance for Musculoskeletal Health (G-MUSC) https://gmusc.com, with one of us (HF) as chair and invited co-chairs joining in 2019; CS (paediatric rheumatology), MD (paediatric orthopaedics) and CT (MSK health promotion). ‘Joining forces’ with PReS has facilitated momentum and growth with global regional representatives and currently > 280 members are signed up to group emails (including doctors, nurses, allied health and representatives from professional societies, industry and research consortia). Communications are facilitated through e-Newsletters, social media and ‘face to face’ meetings at professional gatherings. Our work has no dedicated funding and we acknowledge administrative support from G-MUSC.

**Table:** 

**Table - Aims of the Task Force**	
**To Raise Awareness**	
● About the *many* children and young people around the world with MSK problems	
● About the considerable long-term impact of *untreated* MSK conditions starting in early life: *impact* on young people, their families, carers and society	
● That many conditions are *treatable;* long term disability can be avoided thus reducing ‘cost’ to individuals and society	
**To Identify and Promote tangible exemplar solutions to improve access to ‘right’ care**	
● Models of clinical care and access to medicines	
● Education and training ‘fit for purpose’ and to increase workforce capacity	
● Promote contextually relevant research and wider inclusion	
**To Promote healthy joints and bones**	
● Through lifestyle (e.g. diet, exercise, behaviours)	
● Reduce the risk of injury and reduce levels of obesity	
● Reduce the long term risk of osteoarthritis and osteoporosis	

Our ‘modus operandi’ is ‘working better together’, with focus on learning from each other. It is imperative that we do not ‘reinvent the wheel’; our efforts have been to engage widely, identify exemplar solutions and foster collaborations to extend their benefit to more individuals across different health care contexts. We focus on ‘low hanging fruit’; identifying and supporting exemplars with greatest potential impact and at lower ‘cost’ in terms of resource. Our ‘Call to Action’ encompasses awareness, workforce capacity, models of care, research and policy themes to address the spectrum of MSK health [13]. In many of the exemplar initiatives listed below, Task Force members are working in collaboration to increase their reach and impact.

**Awareness, advocacy and policy:**

- WOrld young Rheumatic Diseases day (WORD) (https://wordday.org); An awareness initiative with global participation and to promote the importance of early diagnosis and access to specialist care.
- The Paediatric Bone and Joint Day (https://www.usbji.org/programs/pediatric-specialty-group/world-pediatric-bone-and-joint-day) highlighting lifestyle changes to optimise MSK health.
- Generation Pep: A programme to encourage physical activity and healthy lifestyle behaviours (https://generationpep.se/sv/).
- Promotion of MSK disability as an important contribution to global NCD burden (e.g. http://www.ncdchild.org)
- The WHO Essential Medicines List (https://list.essentialmeds.org/). An effort is in progress to increase the medications available for paediatric rheumatic diseases [14].
- Juvenile Arthritis Management in Less resourced Countries [15] highlighted need for pragmatic clinical guidelines and research to inform clinical practice in LRIC and a further needs assessment for South East Asia and Asia Pacific countries is underway.
- Wikipedia as an open e-resource with reach to a wide audience. An effort is underway to update relevant pages (e.g https://en.wikipedia.org/wiki/Juvenile_idiopathic_arthritis).

**Models of care, education and training:**

- MiracleFeet: A global programme to develops and support local teams to facilitate diagnosis and management of clubfoot (https://www.miraclefeet.org).
- Project ECHO: A global multidisciplinary educational platform for health care professionals (https://echo.unm.edu/) to enable delivery of high quality clinical care within clinical networks [16].
- RightPath (http://www.rightpath.solutions) is a community-based MSK triage model delivered by paediatric physiotherapists.
- Paediatric Musculoskeletal Matters (PMM) [17] (http://www.pmmonline.org) as a free e-resource with content from global partners, global initiatives for MSK curricula [18, 19] and rheumatology training programmes in countries where specialist care is lacking [19].
- PReS training schemes (e.g. https://www.pres.eu/activities/young-investigators/fellowship-programs.html) open to countries outside of Europe and bursaries for clinicians from LRIC to existing CME events (e.g. EULAR and PReS meetings).

## Conclusions

***“If you want to go fast, go alone. If you want to go far, go together” - African proverb***

The Task Force has gained considerable momentum through amazing engagement and motivation within our global community. More needs to be done and can be done through working better together. Greater awareness and collaboration can enable us to harness existing and emerging knowledge, innovations and technical advances to make real impact and achieve ‘better MSK health for all’. Moving forwards, our ‘Call to Action’ will inform a roadmap to enable real change.

Paediatric Global MSK Task Force: https://gmusc.com/musculoskeletal-problems-in-children-and-young-people/

Twitter @paedmskglobal.

## Data Availability

There is no data generated to analyse.

## Abbreviations

CYP: Children and Young People
EML: Essentials Medicine List
EULAR: European League Against Rheumatism
G-MUSC: Global Alliance for Musculoskeletal Health
LRIC: Low Resource Income Countries
PMM: Paediatric Musculoskeletal Matters
PReS: Pediatric Rheumatology European Society
MRIC: Middle Resource Income Countries
MSK: Musculoskeletal
NCD: Non-Communicable Diseases
WHO: World Health Organisation
